# Effects of action observation therapy and mirror therapy after stroke on rehabilitation outcomes and neural mechanisms by MEG: study protocol for a randomized controlled trial

**DOI:** 10.1186/s13063-017-2205-z

**Published:** 2017-10-04

**Authors:** Tsai-yu Shih, Ching-yi Wu, Keh-chung Lin, Chia-hsiung Cheng, Yu-wei Hsieh, Chia-ling Chen, Chih-jou Lai, Chih-chi Chen

**Affiliations:** 1 Department of Occupational Therapy and Graduate Institute of Behavioral Sciences, College of Medicine, Chang Gung University, 259 Wenhua 1st Rd., Guishan Dist, Taoyuan, Taiwan; 2 Healthy Aging Research Center, Chang Gung University, Taoyuan, Taiwan; 3 Department of Physical Medicine and Rehabilitation, Chang Gung Memorial Hospital, Linkou, Taiwan; 4 School of Occupational Therapy, College of Medicine, National Taiwan University, Taipei, Taiwan; 5 Division of Occupational Therapy, Department of Physical Medicine and Rehabilitation, National Taiwan University Hospital, Taipei, Taiwan; 6 Department of Psychiatry, Chang Gung Memorial Hospital, Linkou, Taiwan; 7 Graduate Institute of Early Intervention, College of Medicine, Chang Gung University, Taoyuan, Taiwan; 8 Department of Physical Medicine and Rehabilitation, Taipei Veterans General Hospital, Taipei, Taiwan; 9 School of Medicine, National Yang-Ming University, Taipei, Taiwan; 10 College of Medicine, Chang Gung University, Taoyuan, Taiwan

**Keywords:** Stroke, Neurorehabilitation, Action observation, Mirror therapy, Magnetoencephalography

## Abstract

**Background:**

Loss of upper-extremity motor function is one of the most debilitating deficits following stroke. Two promising treatment approaches, action observation therapy (AOT) and mirror therapy (MT), aim to enhance motor learning and promote neural reorganization in patients through different afferent inputs and patterns of visual feedback. Both approaches involve different patterns of motor observation, imitation, and execution but share some similar neural bases of the mirror neuron system. AOT and MT used in stroke rehabilitation may confer differential benefits and neural activities that remain to be determined. This clinical trial aims to investigate and compare treatment effects and neural activity changes of AOT and MT with those of the control intervention in patients with subacute stroke.

**Methods/design:**

An estimated total of 90 patients with subacute stroke will be recruited for this study. All participants will be randomly assigned to receive AOT, MT, or control intervention for a 3-week training period (15 sessions). Outcome measurements will be taken at baseline, immediately after treatment, and at the 3-month follow-up. For the magnetoencephalography (MEG) study, we anticipate that we will recruit 12 to 15 patients per group. The primary outcome will be the Fugl-Meyer Assessment score. Secondary outcomes will include the modified Rankin Scale, the Box and Block Test, the ABILHAND questionnaire, the Questionnaire Upon Mental Imagery, the Functional Independence Measure, activity monitors, the Stroke Impact Scale version 3.0, and MEG signals.

**Discussion:**

This clinical trial will provide scientific evidence of treatment effects on motor, functional outcomes, and neural activity mechanisms after AOT and MT in patients with subacute stroke. Further application and use of AOT and MT may include telerehabilitation or home-based rehabilitation through web-based or video teaching.

**Trial registration:**

ClinicalTrials.gov, ID: NCT02871700. Registered on 1 August 2016.

**Electronic supplementary material:**

The online version of this article (doi:10.1186/s13063-017-2205-z) contains supplementary material, which is available to authorized users.

## Background

Stroke is the leading cause of long-term adult disability worldwide [1]. Most patients with stroke experience upper-extremity (UE) motor impairment [2] and show minimal recovery of the affected arm even 6 months after stroke [3]. Due to the potentially severe adverse effects after stroke, it is critical in clinical practice to develop effective and specific stroke interventions to improve arm function and to explore the neural mechanisms involved [4, 5]. Action observation therapy (AOT) and mirror therapy (MT) are two examples of novel approaches concerning stroke motor recovery that are supported by neuroscientific foundations [6, 7]. However, the relative efficacy of AOT versus MT has not been validated in patients with stroke.

AOT is a promising approach grounded in basic neuroscience and the recent discovery of the mirror neuron system (MNS) [6]. AOT commonly includes action observation and action execution and allows patients to safely practice movements and motor tasks. AOT is recommended to help patients with stroke to form accurate images of motor actions [8] and to mediate their motor relearning process after stroke [6]. Researchers have found that AOT can induce stronger cognitive activity than motor imagery in patients with stroke and have suggested that AOT could be an effective approach for patients who have difficulty with motor representation [9]. AOT is a new approach in stroke rehabilitation; therefore, only a few studies have targeted enhancement of UE motor recovery and investigated the effects of AOT in patients with stroke [8, 10–14]. Based on these studies, AOT has been shown to be a beneficial and effective approach to improve patient motor function. However, the heterogeneity of study designs and small sample sizes of the studies lead to no clear conclusions about the efficacy of AOT in stroke rehabilitation.

MT has emerged as another novel stroke-rehabilitation approach during the last decade [15–17]. In this treatment, participants are instructed to move their arms and watch the action reflection of the non-affected arm in the mirror, as if it were the affected one. The process creates the visual illusion of the non-affected arm as the affected arm is normally moving. MT focuses on visual and proprioceptive feedback of the non-affected limb, which may provide substitute inputs for absent or reduced proprioceptive feedback from the affected side of the body [18]. A growing amount of academic literature has demonstrated that patients with stroke gain improvements in motor and daily function, movement control strategies, and activities of daily living [16, 17] after treatment with MT, which supports its use in stroke rehabilitation. In short, MT is potentially a simpler, less expensive, and effective stroke-rehabilitation approach for practical implementation in clinical settings.

Action observation is based on activities of the MNS and mainly involves brain areas of the inferior parietal lobe, inferior frontal gyrus, and ventral premotor cortex [19]. Mirror neurons discharge both during the execution of motor acts or goal-directed actions and during the observation of other people performing the same or similar actions [20]. Experimental studies in healthy adults have demonstrated that the MNS was activated during both the observation and execution of movements, which helped to form new motor patterns during action observation [21–23]. In addition, although positive effects of MT have been demonstrated in patients with stroke [24], there is no consensus about the underlying neural mechanisms of MT. Three hypotheses have been recently proposed to explain the beneficial effects of MT on motor recovery [7]. Accordingly, MT may affect perceptual motor processes via three functional neural networks: (1) activation of brain regions associated with MNS [25, 26], (2) recruitment of ipsilateral motor pathways [27], and (3) substitution of abnormal proprioception from the affected limb with feedback from the non-affected limb [15, 18]. Few AOT and MT neurophysiological or imaging studies have been conducted in patients with stroke. No studies have directly compared and unraveled the similarities or differences in neural plastic changes between AOT and MT in these patients. It is crucial to compare neuroplasticity mechanisms between these intervention regimens to optimize rehabilitative outcomes.

### Objectives

The main purposes of this clinical trial are to (1) compare the immediate and retention treatment effects of AOT and MT on different outcomes with those of a dose-matched control group and (2) explore and compare the neural mechanisms and changes in cortical neural activity associated with the effects of AOT and MT in stroke patients, using magnetoencephalography (MEG).

## Methods/design

### Study design and procedure

**Fig. 1:**
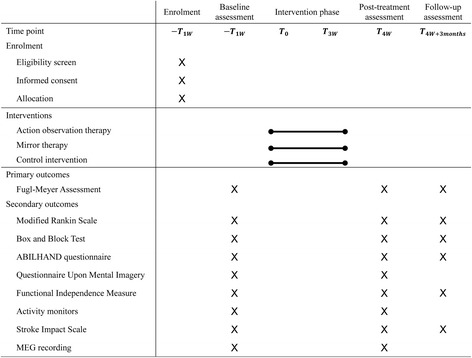
Schedule of enrollment, intervention, and assessment

This study protocol conforms to the Standard Protocol Items: Recommendations for Interventional Trials (SPIRIT) guidelines (see Additional file 1). This three-arm, single-blind, randomized controlled trial will investigate the treatment effects of AOT and MT versus a control intervention and accords with the SPIRIT Figure (Fig. 1). An estimated 90 patients with subacute stroke will be recruited to participate in this study. Each participant will receive 15 training sessions for 3 weeks. Licensed occupational therapists will be well trained in treatment protocols and study procedures to ensure the consistency of intervention delivery. The therapists were not blinded to the group assignment, given the nature of the intervention. In addition, all outcome measures will be administered to the patients by the same rater, who will be blinded to the subject’s group. Three evaluation time points will be used: baseline, immediately after 3 weeks of treatment, and 3 months after treatment. Prior to administration of outcome measures, a blinded rater will be trained on how to appropriately administer these assessments. Rater competence will be evaluated before proceeding to the trial.

### Patient inclusion and exclusion criteria

The inclusion criteria of the participants will be: (1) diagnosed as having a unilateral stroke, (2) 1 to 6 months after stroke onset, (3) from 20 to 80 years of age, (4) a baseline score of the Fugl-Meyer Assessment (FMA) of 20 to 60 [28], (5) able to follow the study instructions (measured by the Taiwan version of the Montreal Cognitive Assessment) [29], and (6) capable of participating in therapy and assessment sessions. The exclusion criteria will be: (1) global or receptive aphasia, (2) severe neglect, and (3) major medical problems or comorbidities that influenced UE usage or cause severe pain.

In addition, if patients have no metal implants, no head traumas or neurosurgical operations, and can perform MEG motor tasks, they can additionally participate in the MEG assessment. For the MEG portion of the study, we expect to recruit 12 to 15 patients in each group. Patients can still participate in the study, receiving rehabilitation therapy and clinical evaluations, even if they do not meet the MEG eligibility criteria, or are not willing to participate in the MEG study. All participants must provide written informed consent before entering the study.

### Randomization

The participants will be randomly allocated to one of the three treatment groups in a 1:1:1 ratio after baseline evaluation. The participants will be pre-stratified on the basis of the severity of UE motor deficits (FMA score: 20 to 40 (moderate-to-severe) versus 40 to 60 (mild-to-moderate) [28]) and side of lesion (right versus left) to assure baseline equivalence between the groups. The random assignments will be performed online using a web-based randomization tool (freely available at https://www.randomizer.org/). A research assistant who will not be involved in outcomes assessment or screening of subjects will independently manage the randomization procedure according to allocation concealment.

### Intervention

All patients will receive treatment for 60 min per day, 5 days per week for 3 weeks (15 sessions). For all three interventions, the therapists will provide verbal instructions, cues, feedback, and help, when needed.

#### Action observation therapy (AOT)

The AOT group will observe UE movement video clips and practice the movement simultaneously or sequentially. The video movements will be displayed from a first-person perspective to maximize corticomotor excitability [30]. The movements and tasks in the video will be performed by young, healthy adults, either men or women. Based on findings in the academic literature and clinical expertise, the following common categories of movements and tasks will be included in this study: (a) active range of motion (AROM) exercises (10–15 min), (b) reaching movement or object manipulation (15–20 min), and (c) UE functional tasks (30 min).

**Fig. 2:**
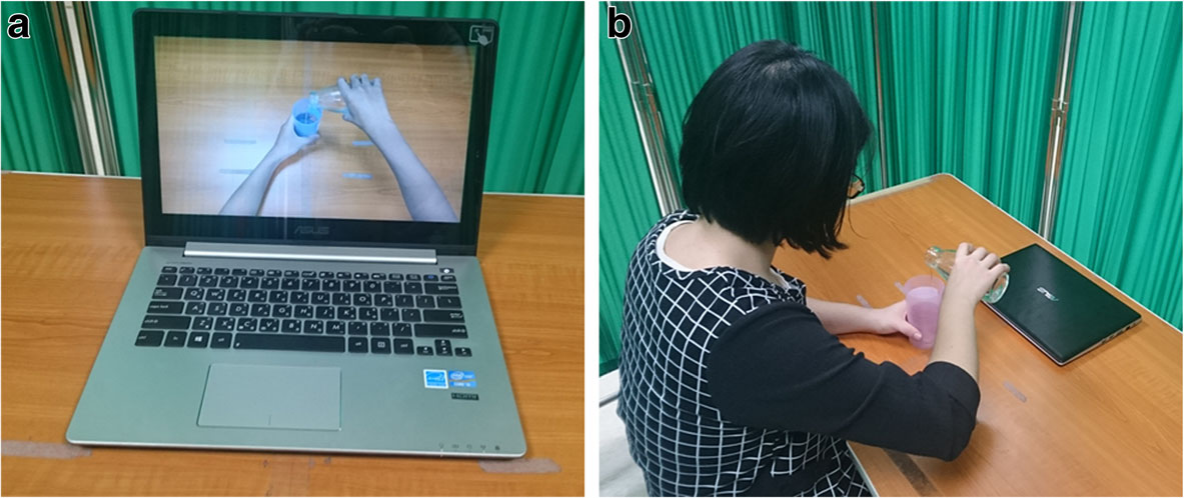
Action observation therapy. **a** Observation of task. **b** Execution of task

During phase 1, the patients will observe AROM exercises through video clips, and move both of their UE simultaneously. In phase 2, the patients will observe one reaching or object manipulation task, depending on the patient’s motor ability, for 2 min through a video clip, and will be required to execute and practice the movements that they observed for 3 min. This sequence will be repeated three times. Phase 3 will contain one functional task in each session, starting with easy then more complex tasks. Each functional task will be divided into three motor acts (Fig. 2). For example, the action of drinking bottled water will be decomposed into the following three motor acts: (1) reaching and grasping the bottle, (2) opening the bottle cap, and (3) picking up the bottle. After observing a motor act in a video clip for 2 min, the patients will be asked to execute the actions that they observed, for 3 min. For the next 15 min, the patients will observe the functional task as a complete action for 2 min and then execute the entire task for 3 min which will be repeated three times. Examples of functional tasks are reading a magazine, folding a towel, drinking water, and wiping a table.

#### Mirror therapy (MT)

**Fig. 3:**
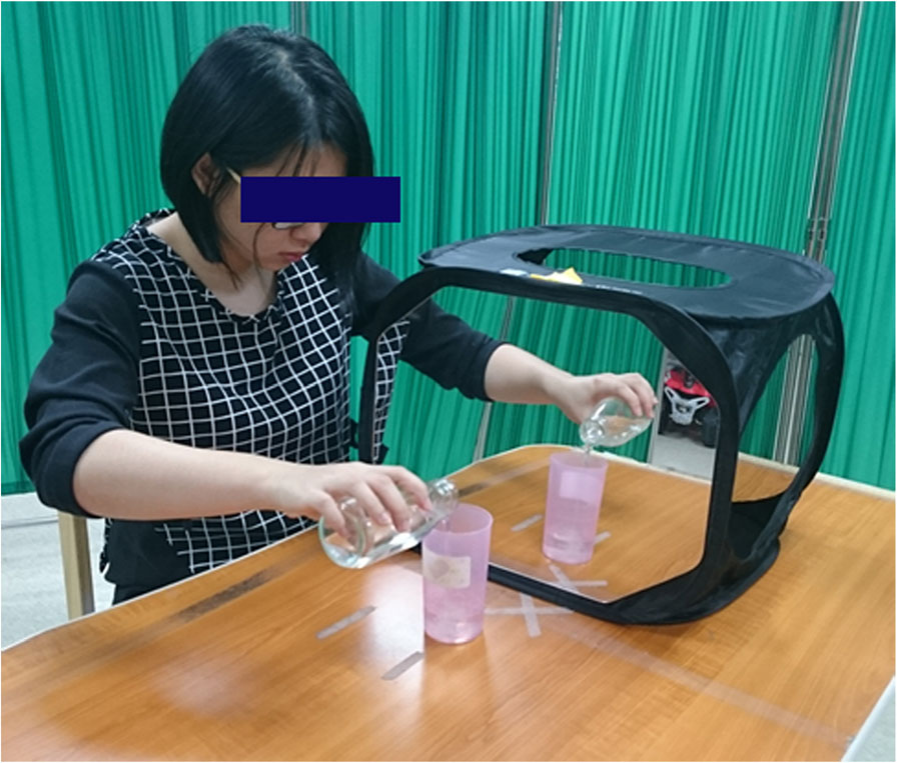
Mirror therapy

The MT group will receive 60 min of UE training in a mirror box. A portable and foldable mirror box that can be easily carried will be used. During the mirror practices, the patient will sit close to a table, on which the mirror box will be placed at their mid-sagittal plane. The affected arm will be placed behind the mirror, and the unaffected arm will be in front of the mirror. The patient will be instructed to watch the image of their unaffected arm in the mirror (Fig. 3). During MT, the participant will be encouraged to actively move their affected arm and hand concurrently with the mirror reflection of the unaffected arm and hand as much as they can. If necessary, the therapist will assist the participant in moving the affected hand, to synchronize the movement with the unaffected hand. MT treatment activities will also include AROM exercises (10–15 min), reaching movement or object manipulation (15–20 min), and functional tasks practice (30 min) in a mirror box.

#### Control group – customary bilateral UE training

**Fig. 4:**
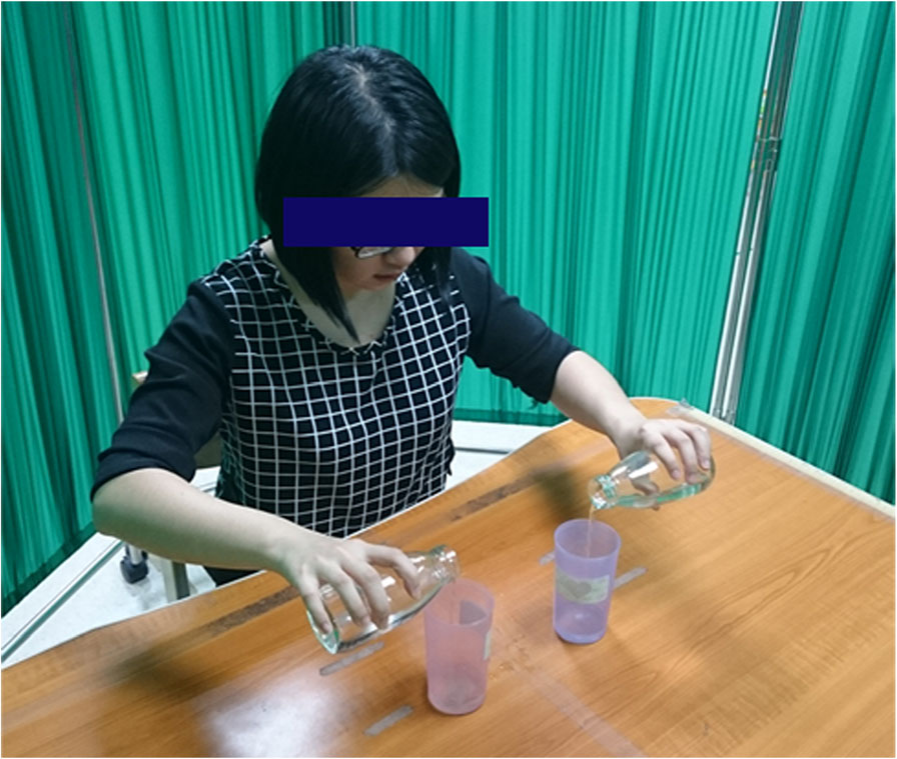
Control treatment

The control group will receive bilateral UE training without watching videos or performing activities in a mirror box. The same categories of movements and tasks as those in the AOT and MT groups will be used in the control group, but neither videos nor a mirror box will be provided. During training, the patients will be required to move both arms and hands simultaneously (Fig. 4). The levels of movement or task difficulty can be adjusted depending on the patient’s level of motor ability and progress.

### Outcome measures

Clinical outcome measurements will be administered to the participants at baseline, immediately after 3 weeks of treatment, and 3 months after treatment. The primary outcome will be the FMA score. Secondary outcomes will include the modified Rankin Scale (mRS), the Box and Block Test (BBT), the ABILHAND questionnaire, the Questionnaire Upon Mental Imagery (QMI), the Functional Independence Measure (FIM), activity monitors, and the Stroke Impact Scale (SIS) version 3.0.

#### Primary outcomes

The upper-limb subscale of the FMA will be used to evaluate motor impairments [28]. The 33 items assess movements, reflexes, and coordination of the shoulder, elbow, forearm, wrist, and hand. The total score ranges from 0 to 66, with a higher score indicating less motor deficit. The psychometric properties of the FMA have been well established in patients with stroke [31].

#### Secondary outcomes

The mRS will be used to assess the degree of stroke disability in this study. The mRS was found to be valid and reliable in stroke patients [32]. The mRS scores range from 0 to 5. Higher scores indicate more severe disability. Favorable outcomes of patients are defined as the mRS score of ≤ 2 which indicates no or slight disability [33].

The BBT is a measure of hand dexterity with satisfactory reliability and validity in patients with stroke [34, 35]. The BBT to be used in this study contains 150 colored wooden cubes in a box with two compartments. The participants will be instructed to use their affected hand to move as many blocks as possible one-by-one from one compartment to the other within 1 min. The number of blocks transferred by the affected hand is counted as the score of the BBT.

The ABILHAND questionnaire is self-reported and assesses patients’ perceived difficulty in performing daily activities that require the use of bilateral UE [36]. It contains 23 daily activities measuring bimanual ability and is rated on a 3-point scale (0 = impossible, 1 = difficult, 2 = easy). Its psychometric properties have been validated in patients with stroke [37].

The short-form of the QMI will be applied to assess the ability of the patient’s mental imagery. The 35-item questionnaire consists of seven domains of sensory modalities [38]. The score of each item ranges from 1 (perfectly clear) to 7 (no image present at all). A lower score indicates better mental imagery ability. The test-retest reliability of QMI has been validated [39].

The FIM is a scale frequently used to assess basic activities of daily function. It consists of 18 items with a total score ranging from 0 to 126 [40]. The FIM includes six subscales: self-care, sphincter control, transfer, locomotion, communication, and social cognition ability. A higher score indicates less disability for basic daily function. The FIM is an assessment with good reliability, validity, and responsiveness [41, 42].

The ActiGraph GT3X^+^ accelerometers (ActiGraph, Pensacola, FL, USA) will be used to provide an objective measure of the amount of using the affected arm in the patients’ real-life environments [43]. The participants will wear the accelerometers on each wrist for three consecutive days before and after treatment. The main outcome parameters will be the amount of physical activity (counts/min), activity intensity, and energy expenditure in real life. The accelerometer-based devices are commonly used to monitor physical activity in stroke patients [44].

The SIS version 3.0 is a patient-reported outcome to evaluate function, participation, and health-related quality of life of stroke survivors with sound psychometric properties [45]. It consists of 59 items, with a higher score indicating better function and greater participation.

#### MEG recordings

The whole-head 306-channel MEG (Vectorview, Elekta Neuromag, Helsinki, Finland) will be used in this study. The data from planar gradiometers of this device, which detect the largest signal directly above the activated cerebral areas, will be analyzed. The MEG signals will be digitized at a sampling rate of 500 Hz, with an online bandpass of (0.1, 200) Hz. An interval of 0.5 s, including a pre-stimulus baseline of 0.1 s, will be evaluated. Epochs contaminated by prominent electrooculogram signals (>300 μV) and MEG artifacts (> 3000 fT/cm) will be automatically excluded from the averaging [46]. At least 100 artifact-free evoked responses will be averaged online. For all tasks, the median nerve of the affected hand will be stimulated with an interstimulus interval of 1.5 s by an electrical stimulator (Konstantstrom Stimulator, Schwind, Erlangen, Germany). Stimulus intensity will be set at 20% above the motor threshold, to elicit a visible twitch of the abductor pollicis brevis muscle. In the sensory task, the subject will be asked to sit comfortably and look at a fixed position in front of them. For the motor tasks, study participants will be instructed to perform the following tasks: resting, executing movement, watching a video and executing movement simultaneously, video-watching first and then executing movement, and executing movement with and without a mirror reflection of the unaffected arm’s movement. For all conditions, the beta event-related synchronization and beta event-related desynchronization will be extracted as electrophysiological indices of motor cortical activation or inhibition. The electromyography signals from muscles will be used to determine the onset of the movements.

### Monitoring potential adverse events

Patient-reported fatigue and pain will be measured by use of an item in the Numerical Rating Scale, on a 10-cm vertical line supplemented with the Face Rating Scale. The therapist will ask the patient to rate the severity of their pain and fatigue during the treatment on a scale from 0 (no pain or no fatigue) to 10 (unbearable pain or exhaustion) on the first and final treatment days. In addition, patients will also be instructed to report any other adverse effects to the therapist or the investigator whenever they occur.

### Sample size calculation

To estimate the sample size, a-priori power analysis is performed on the data from previous AOT [8, 11] and MT literature [17, 24] in stroke rehabilitation. Based on the previous data of the treatment effects on motor function outcomes (e.g., FMA), an effect size (*d*) of 0.60 to 0.95 is expected which corresponds approximately to an effect size (*f*) of 0.30 to 0.50 for a study design of three-group comparisons. An estimate of sample size requirements for each group in a three-group study design, given a power of 0.80 and a two-sided type-I error of 0.05, will be in the range of 21 to 36 patients. We therefore plan to recruit 30 participants in each group (a total of 90) in this study.

### Data analysis

An intention-to-treat analysis will be performed. Two-way repeated measures analysis of variance (ANOVA) will be used to evaluate the treatment effects among the three treatment groups at the three assessments from different time points. The between-subject factor will be the group and the within-subject factor will be the time. The treatment efficacy will also be examined by determining the number of participants whose change score reaches the minimal clinically important difference (MCID) in the clinical outcomes, by using a chi-square test. The MCID values will be adopted either for the established values of outcomes or 10% of the total scores when it has not been established. A *p* value < 0.05 will be considered as indicating statistical significance.

## Discussion

The study aim is to investigate the treatment efficacy and mechanisms of neural activity of AOT and MT and compare them with those of the control intervention for patients with stroke. AOT and MT are two novel and promising approaches for enhancement of stroke motor recovery through different afferent inputs and patterns of visual feedback. AOT focuses on the observation of actual actions of another person and execution of what is seen, whereas MT emphasizes observing the mirror reflection of arm movements in the non-affected arm, as if it were the affected one. AOT and MT aim to prime the motor system through the MNS for promoting neuroplasticity and motor function of stroke patients; however, their effectiveness in stroke rehabilitation is equivocal and warrants investigation.

While translating research evidence into clinical practice, patients can receive recommended rehabilitation care and therapy. After the study has been completed, and if positive results are achieved, we will provide scientific evidence of treatment effects and neural activity changes after AOT and MT. Based on the overall study results, our approaches might become new add-on features of modern neurorehabilitative treatments, and could potentially be used in rehabilitation settings. Future applications of AOT and MT may include telerehabilitation or home-based rehabilitation through web-based or video teaching.

## Trial status

At the time of submission, the recruitment of study participants is ongoing.

## Additional file

Additional file 1: SPIRIT 2013 Checklist: recommended items to address in a clinical trial protocol and related documents. (PDF 60 kb)

## Abbreviations

AOT: Action observation therapy
AROM: Active range of motion
BBT: Box and Block Test
FIM: Functional Independence Measure
FMA: Fugl-Meyer Assessment
MCID: Minimal clinically important difference
MEG: Magnetoencephalography
MNS: Mirror neuron system
mRS: Modified Rankin Scale
MT: Mirror therapy
QMI: Questionnaire Upon Mental Imagery
SIS: Stroke Impact Scale
UE: Upper extremity
